# A sliding mode control of the bearingless permanent magnet slice motor for the blood pump based on the GAPSO

**DOI:** 10.1038/s41598-023-45891-w

**Published:** 2023-11-02

**Authors:** Xin Liu, Hongyi Qu, Lingwei Meng, Chuangxin Huang, Qi Chen, Qiuliang Wang

**Affiliations:** 1 Ganjiang Innovation Academy, Chinese Academy of Sciences, Ganzhou, 341000 Jiangxi People’s Republic of China; 2 Department of Automation, University of Science and Technology of China, Hefei, 230026 Anhui People’s Republic of China; 3 Institute of Electrical Engineering, Chinese Academy of Sciences, Beijing, 100190 People’s Republic of China

**Keywords:** Cardiology, Engineering

## Abstract

In this paper, we proposed a sliding mode control method for the bearingless permanent magnet slice motor for the blood pump based on the genetic particle swarm algorithm, which aims to solve the problems of strong coupling, strong interference, nonlinearity and uncertainty. Firstly, the mathematical model of rotor torque and suspension force of the bearingless permanent magnet slice motor is established. Secondly, the structure of sliding mode observer is deduced by designing sliding mode surface and control law. And, the performance parameters of sliding mode observer are optimized by the genetic particle swarm optimization algorithm. Thirdly, electromagnetic torque and suspension force control under this control method is studied by Simulink. Finally, the control method is applied to the control of the blood flow of the blood pump, and the rotation speed can effectively control the blood flow. The results indicate that compared with PID control and traditional sliding mode control methods, the sliding mode control method optimized by the genetic particle swarm optimization algorithm greatly improves the control performance of bearingless permanent magnet slice motor. The results show that the blood flow can meet expectations with a small error, which fully meets the blood perfusion requirements of the blood pump.

## Introduction

The high mortality rate of heart failure and the number of diseases that continuance to increase are a common problem facing the medical community today. Heart transplantation is the best way to treat heart failure, but the number of portable hearts transplanted every year is very small and it is also greatly affected by the matching of supply and demand^1–3^. Since its inception, the blood pump has been widely used in cardiovascular treatment, assisting the damaged heart to complete the pumping of blood, which has become the last hope for prolonged survival of many patients with heart failure^4,5^. Patients with severe heart failure might use this device to assist the blood circulating throughout their bodies. A complete mechanical heart can lengthen life and help people live better. It is worth mentioning that bleeding, infection and organ failure are among the complications that might result following a implantation of a blood pump.

Bearingless permanent magnet slice motor (BPMSM) is a magnetic levitation machine that allows the rotor to stably levitate at 5 degrees of freedom. Compared to other motors, on the one hand, it does not have mechanical bearings, which can greatly reduce the blood compatibility issues caused by friction and wear. On the other hand, its volume can be made very small and its energy consumption is very low. It is precisely because of these two advantages that it has gradually become the power source of the new generation of blood pump^6,7^. The clinical trials showed that the blood pump system driven with BPMSM has high reliability and durability, and has an extremely low probability of hemolysis and coagulation accidents, which makes the BPMSM type of the blood pump very competitive^8,9^. The control goal of BPMSM for the blood pump is to suspend the rotor impeller in the pump room stably, and the high-speed rotation assists the natural heart blood beating to meet the blood perfusion needs of the human circulatory system. However, the control system of BPMSM has the disadvantages of parameter uncertainty, complex temporal degeneration and system nonlinearity^10^. It is worth mentioning that its control performance directly determines the suspension performance and rotational performance of the blood pump. Therefore, it is crucial to study the blood pump control technology with BPMSM.

Many scholars have explored the control methods of BPMSM. Reference^11^ proposes a direct torque direct suspension force control method of BPMSM. The direct torque control model is first established and then applied to the suspension module, and then the direct suspension force control model is based. Finally, the effectiveness of the method is verified by simulation. Reference^12^ designs a BPMSM control strategy based on a phase-locked loop flux linkage observer. The simulation and experimental results show that this strategy can meet the control accuracy required by the target and has good stability. In recent years, the sliding mode control method (SMC) has been widely used in motor control due to its advantages of fast response, strong anti-interference ability, and easy implementation^13^. Reference^11^ reported a sensorless control method of bearingless permanent magnet synchronous motor based on the BP neural network. The results show that the method can accurately estimate the position and speed of the rotor, and the identification accuracy is high. Reference^14^ proposes an improved sliding mode control method and applies it to the control of the speed and radial displacement of the BPMSM rotor. It is found that the control effect of this method is remarkable through simulation and experiments. Reference^15^ proposes a BPMSM neural sliding mode control method, which realizes the decoupling control of the motor through nonlinear differential geometry. The application of the sliding mode control method in BPMSM for the blood pump is seldom, and there are only a few reports. Reference^16^ proposes a tracking control method based on sliding mode control, which uses the blood pump flow pulsation as a feedback parameter to adjust the average pump flow. The speed and robustness of the proposed method are verified by simulation.

Although the sliding mode control is effective in BPMSM, it also has defects such as the instability of the sliding mode control system due to the high-frequency chattering. The main reason is that the parameters in the sliding mode function are improperly set. Particle swarm optimization (PSO) algorithm is an intelligent optimization algorithm, widely used in performance parameter optimization. It can effectively solve the problem of parameter optimization of sliding mode control system in the BPMSM for the blood pump^17,18^. Genetic algorithm-particle swarm optimization (GAPSO) is an improved algorithm of particle swarm optimization, which can improve the defect of traditional particle swarm algorithms that are prone to falling into local optima.

This paper proposes a sliding mode control method for the blood pump motors based on the GAPSO algorithm. In "Mathematical model of the BPMSM" section, the torque expression and the suspension force expression of the BPMSM are established. In "Design of the BPMSM sliding mode controller for the blood pump" section, the structure of the sliding mode observer is deduced by designing the sliding mode surface and the control law, and the performance parameters of the sliding mode observer are optimized by using the GAPSO algorithm. In "Simulation study" section, the electromagnetic torque and suspension force control under the control method are studied through Simulink simulation. In "Simulation experiments" section, the control method is applied to the control of the blood flow of the blood pump, and its effectiveness is verified. In “Conclusion” section, conclusions are drawn.

## Mathematical model of the BPMSM

**Figure 1:**
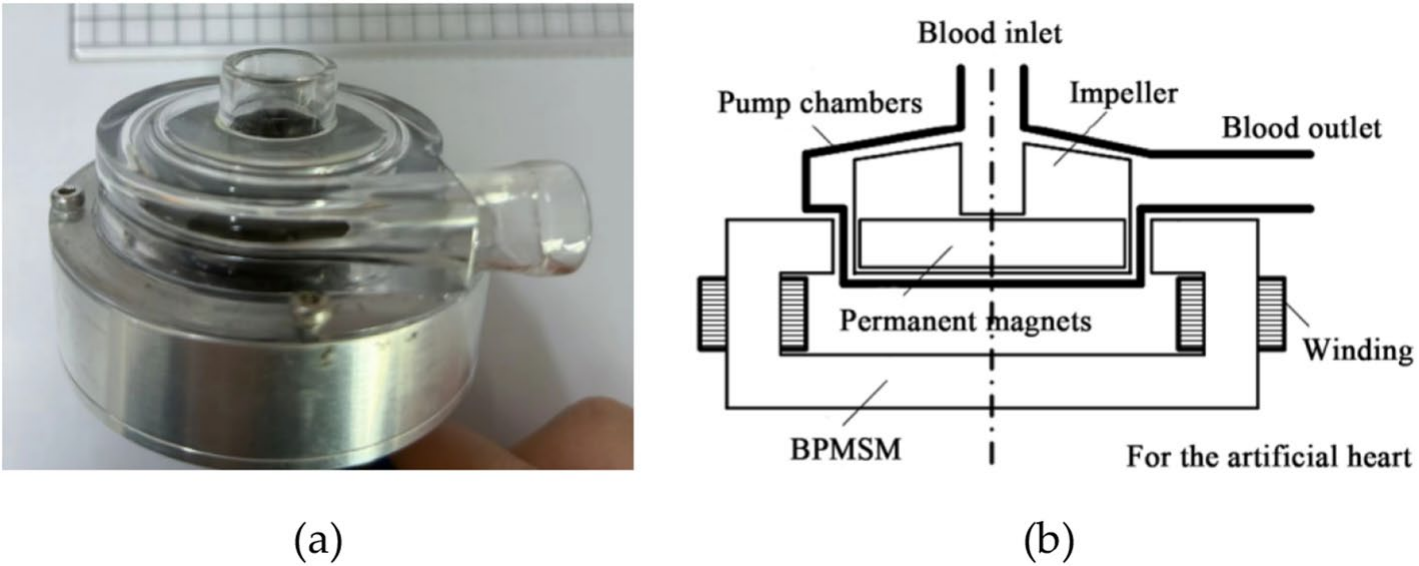
The magnetic levitation blood pump: (**a**) the object picture; (**b**) the schematic diagram.

This research focuses on the research of a magnetic levitation blood pump independently developed in the laboratory, where the impeller is driven by the rotor of a bearingless permanent magnet thin film motor inside the pump to complete rotation. While rotating, the impeller can still be stably suspended in the pump chamber, relying on a hybrid suspension mechanism combining electromagnetic suspension and permanent magnetic suspension. The physical and schematic diagram of the magnetic levitation blood pump pump is shown in Fig. 1.

**Figure 2:**
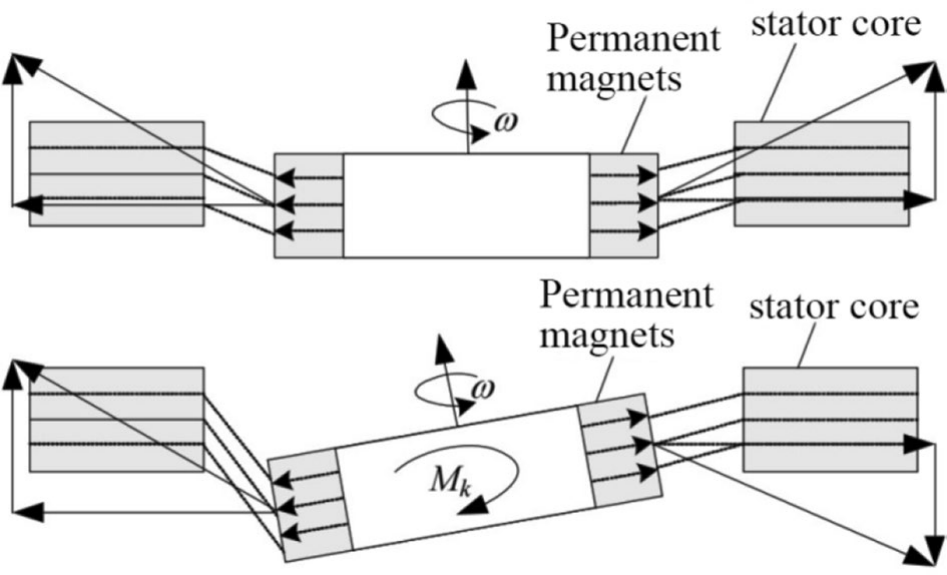
Passive suspension schematic diagram.

Among Fig. 1, the passive suspension of axial permanent magnet refers to the suspension at three degrees of freedom: axial offset, axial left torsion, and axial right torsion. To achieve this suspension, the rotor needs to be made into a thin sheet shape when designing a bearingless permanent magnet motor, so that its axial length is much lower than its radial length. When the rotor is disturbed and deviates from the center of the pump chamber, the magnetic field line between the rotor and stator will become longer, and at this time, a tensile force will be applied to pull the rotor back to its equilibrium position. The schematic diagram of passive suspension is shown in Fig. 2^19^. This relationship allows the rotor to be maintained in the center position without external force, rather than hitting the inner wall of the pump chamber.

The fact that passive suspension is simpler than active suspension and does not require the design of suspension windings and feedback circuits, so this study does not describe this not much. The focus is on the active control of radial suspension force and torque. A BPMSM is essentially a permanent magnet synchronous motor with a thin rotor rotation. Therefore, in torque control, we adopted the most commonly used rotor field oriented vector control for permanent magnet synchronous motors. Because this control strategy is simple and reliable, and the torque characteristics of the motor are good under this control method.

**Figure 3:**
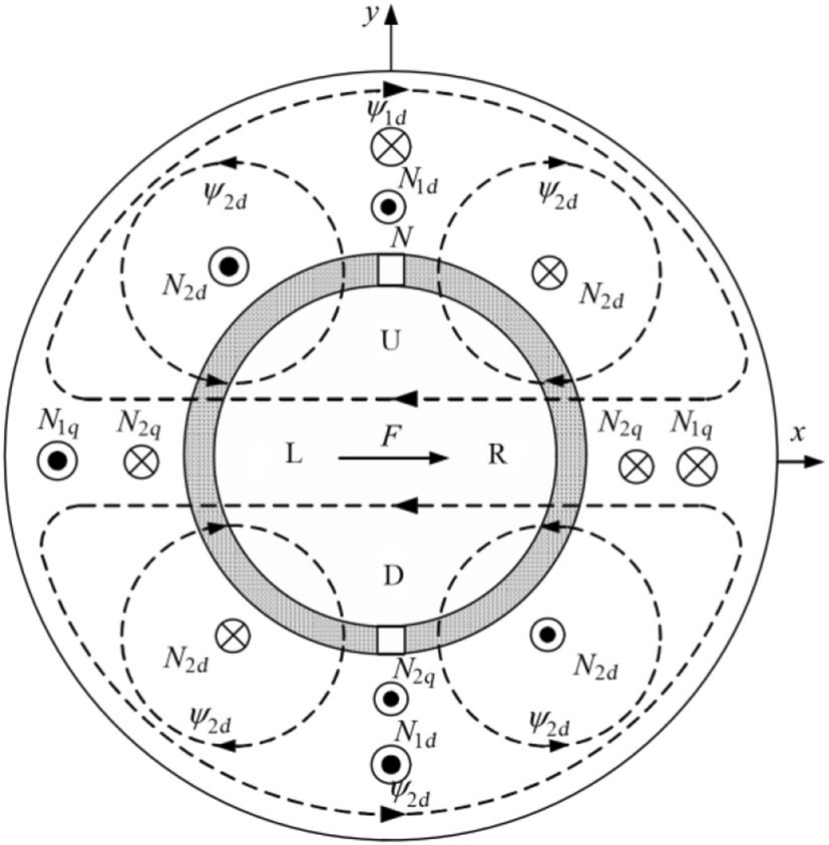
The schematic diagram of the generation of levitation force.

The motor rotor is stably suspended radially through active electromagnetic control, which is the result of the superposition of two magnetic fields. For example, when the rotor has an offset in the *x*-negative direction of the radial plane, the displacement sensor will send the offset to the controller. At this time, the suspension winding will be energized to generate a magnetic field. The superposition of this magnetic field with the magnetic field of the permanent magnet will make the magnetic density in the *x*-positive direction greater than that in the *x*-negative direction, which will generate a magnetic pull force in the *x*-positive direction, pulling the rotor in the *x*-negative direction back to its equilibrium position. If the rotor is offset at any point on the radial direction other than the *x* or *y* axis, the point can be projected onto the *x* or *y* axis and controlled separately. Figure 3 shows the principle of electromagnetic active suspension^13^. The biggest difference between electromagnetic active suspension and permanent magnet passive suspension is that the former is manually controlled and requires power, while the latter is determined by the structure and load of the permanent magnet. Generally, comprehensive consideration and verification are required during design.

The mathematical expression of the controllable radial suspension force *Fx'*, *Fy'*, and the torque *Te* of the BPMSM can be derived as follows^20^:

1 $$ \left\{ {\begin{array}{*{20}c} {F_{{\text{x}}}^{\prime} = k_{m} I_{1f} I_{2} \cos (u - \lambda_{1} )} \\ {F_{{\text{y}}}^{\prime} = k_{m} I_{1f} I_{2} \sin (u - \lambda_{1} )} \\ \end{array} } \right.\;,\;k_{m} = \frac{9}{2}\frac{{\mu_{0} lrN_{1} N_{2} k_{d1} k_{d2} }}{{\pi \delta_{0}^{2} }} $$

2 $$ \left\{ {\begin{array}{*{20}l} {T_{{\text{e}}} = p_{1} \psi_{F} I_{1q} } \hfill \\ {\frac{{di_{1d} }}{dt} = - \frac{{R_{1} }}{{L_{1d} }}I_{1d} + \frac{{u_{1d} }}{{L_{1d} }} + p_{1} \omega I_{1q} } \hfill \\ {\frac{{di_{1q} }}{dt} = - \frac{{R_{1} }}{{L_{1q} }}I_{1q} + \frac{{u_{1q} }}{{L_{1q} }} - p_{1} \omega I_{1d} - \frac{1}{{L_{1q} }}p_{1} \omega \psi_{F} } \hfill \\ {\frac{d\omega }{{dt}} = \frac{1}{J}(T_{e} - F\omega - T_{m} )} \hfill \\ \end{array} } \right. $$

where *u* is the initial phase angle, *λ*_1_ is the initial phase angle of the suspension winding current, *I*_1d_ and *I*_1q_ are the components of the torque winding current on the *d* and *q* axes respectively, *ω* is the rotor speed, *L*_1d_ and *L*_1q_ are the components of the equivalent inductors in the *d* and *q* axes respectively, *R*_1_ is the equivalent resistance on the torque winding, *J* is the moment of inertia, *F* is the load force, *T*_m_ is the load torque, *k*_*m*_ is the current stiffness coefficient, *k*_*n*_ is the displacement stiffness coefficient, *μ*_0_ is the vacuum permeability, *r* is the radius of the motor rotor, *N*_1_ and *N*_2_ are the number of turns of the torque winding and the suspension winding respectively, *l* is the effective length of the rotor, *δ*_0_ is the average length of the air gap without eccentricity, *p*_1_ is the number of pole pairs of the torque winding, *ξ* is the angle between the torque winding flux linkage and the rotor flux linkage, *ψ*_F_ and *ψ*_1_ are the flux linkage amplitudes of the permanent magnet and the torque winding, respectively, *k*_d1_ and *k*_d2_ are the fundamental wave winding distribution coefficient, *ξ* is the equivalent torque winding inductance.

Using the coordinate transformation, Formula (1) can be converted into a formula under the dq coordinate system as follows:

3 $$ \left[ {\begin{array}{*{20}c} {F_{{\text{d}}}^{\prime} } \\ {F_{{\text{q}}}^{\prime} } \\ \end{array} } \right] = k_{m} \left[ {\begin{array}{*{20}c} {I_{f} } & {I_{1} } \\ {I_{1} } & {I_{f} } \\ \end{array} } \right]\left[ {\begin{array}{*{20}c} {I_{2d} } \\ {I_{2q} } \\ \end{array} } \right] $$

where *F*_d_^′^ and *F*_q_^′^ are the components of the controllable radial suspension force on the *d* and *q* axes respectively, *I*_*f*_ and *I*_1_ are equivalent excitation current of the permanent magnet and torque winding respectively, *I*_2d_ and *I*_2q_ are the components of the suspension winding current on the *d* and *q* axes, respectively.

The establishment of torque model and suspension force model is the first step, the next step will design a sliding film controller for that mathematical model.

## Design of the BPMSM sliding mode controller for the blood pump

Sliding mode control includes sliding mode surface design and control law design. The quality of the sliding surface design directly affects the convergence performance of the control system, while the control law design influences the performance of the approach motion.

To prevent singularity, the terminal sliding mode surface was changed to a universal sliding mode surface witha nonlinear function. The non-singular slide mode surface is selected as the BPMSM slide mode controller for the blood pump, which can solve the singular problem in the sliding mode control and has a good convergence performance^21^. With the formula as follows:

4 $$ s = x_{{1}} + \frac{1}{\beta }x_{2}^{p/q} $$

where *β* > 0, *p* > *q*, *p* and *q* are positive odd number, and 1 < *p*/*q* < 2.

Then the time for the control system to reach the equilibrium state from any initial state can be expressed as follows:

5 $$ t_{s} = \frac{p}{{\beta^{q/p} (p - q)}}X(0)^{(p - q)/p} $$

The next step is to design the control law. The design of the control law requires meeting the sliding mode arrival condition, and the sliding mode global arrival condition is as follows:

6 $$ s(X)\dot{s}(X) < 0 $$

Equation (6) can also be regarded as a generalized sliding mode condition. In practical applications, in order to ensure that the state is reachable in finite time, Eq. (6) is usually transformed as follows:

7 $$ s(X)\dot{s}(X) < - \eta ,\;\eta > 0 $$

Exponential Reaching Law (ERL) is one of the strategies mentioned for reducing chattering in sliding mode. By using an exponential modification, ERL might increase chattering alleviation for a given reaching speed as compared to a constant reaching law. Therefore, this paper adopts the most commonly used exponential reaching law with better performance, and its expression is as follows:

8 $$ \dot{s} = - \varepsilon {\text{sgn}} (s) - ks\;\;\;\;\varepsilon > 0\;\;\;k > 0 $$

where *k* and *ε* are two key performance parameters of the control law. The larger *k* is, the faster the system can approach the switching surface, and the lowering of *ε* can improve the occurrence of system chattering. Sgn(*s*) is a symbolic function whose expression is:

9 $$ {\text{sgn}} (s) = \left\{ {\begin{array}{*{20}c} {1\;\;\;\;\;s > 0} \\ { - 1\;\;\;\;\;s < 0} \\ \end{array} } \right. $$

Nonlinear dynamical systems, which describe changes in variables over time, might look chaotic, unexpected, or counterintuitive when compared to simpler linear systems. The BPMSM control system for the blood pump is a second-order nonlinear dynamic system, and its general expression is as follows^22^:

10 $$ \left\{ {\begin{array}{*{20}l} {\dot{x}_{{1}} = x_{2} } \hfill \\ {\dot{x}_{2} = f(X) + b(X)u + g(t)} \hfill \\ \end{array} } \right. $$

where $X{ = [}x_{{1}} {,}x_{{2}} {]}^{{\text{T}}}$ is the system state variable, *u* is the control input, $g(t)$ is the external disturbance.

By analyzing Formula (4), Formula (7), and Formula (10), the following formula can be obtained:

11 $$ u = - {\text{b}}^{{{ - }1}} (X)[f(X) + \beta \frac{q}{p}x_{2}^{{2 - \frac{p}{q}}} + l_{g} \cdot {\text{sgn}} (s) + \;\varepsilon \cdot \frac{1}{{1 + c\left\| X \right\|_{1} }}{\text{sgn}} (s) + (k + c\left\| X \right\|_{1} )s] $$

where $\beta ,\;q,\;p,\;l_{g} ,\;\;\varepsilon ,\;k,\;c$ are performance parameters of the controller, and the quality of their settings directly affects the control performance and chattering size of the controller. However, conventional sliding mode controllers often achieve poor results due to improper parameter settings, so optimization algorithms need to be used to optimize them.

### Design of the slide mode controller for torque

Assuming that the given speed of the BPMSM rotor is ω* and the actual speed is *ω*, then the state variable of the speed error can be listed as:

12 $$ \left\{ {\begin{array}{*{20}l} {e_{\omega 1} = \omega^{*} - \omega } \hfill \\ {e_{\omega 2} = e_{\omega 1}^{\prime } = \dot{\omega }^{*} - \dot{\omega }} \hfill \\ \end{array} } \right. $$

Simultaneous formula (2) and formula (9), the following formula can be obtained:

13 $$ \dot{e}_{\omega 2} = \ddot{\omega }^{*} - \ddot{\omega } = \ddot{\omega }^{*} - \left (\frac{{P_{1} \psi_{f} }}{J}i_{1q} - \frac{F}{J}\dot{\omega } - \frac{1}{J}\dot{T}_{m}\right ) = - \frac{F}{J}\ddot{e}_{\omega 2} - \frac{{P_{1} \psi_{f} }}{J}i_{1q} + \frac{1}{J}\dot{T}_{m} + \ddot{\omega }^{*} + \frac{F}{J}\dot{\omega }^{*} $$

In summary, the state equation of the rotor speed error system can be expressed as:

14 $$ \left\{ {\begin{array}{*{20}l} {\dot{e}_{\omega 1} = e_{\omega 2} } \hfill \\ {\dot{e}_{\omega 2} = - \frac{F}{J}\ddot{e}_{\omega 2} - \frac{{P_{1} \psi_{f} }}{J}i_{1q} + \frac{1}{J}\dot{T}_{m} + \ddot{\omega }^{*} + \frac{F}{J}\dot{\omega }^{*} } \hfill \\ \end{array} } \right. $$

### Design of the slide mode controller for suspension force

Assuming that the given offset of the BPMSM rotor in the *x* direction is x*, and the actual offset is *x*, the state variable of the offset error in the *x* direction is:

15 $$ \left\{ {\begin{array}{*{20}l} {e_{x1} = x^{*} - x} \hfill \\ {e_{x2} = e_{x1}^{\prime } = \dot{x}^{*} - \dot{x}} \hfill \\ \end{array} } \right. $$

Simultaneous formula (1) and formula (9), the following formula can be obtained:

16 $$ \dot{e}_{x2} = \frac{{k_{s} }}{m}e_{x1} - \frac{1}{m}F_{cx} + \ddot{x}^{*} - \frac{1}{m}k_{ecc} x^{*} + \frac{1}{m}F_{xr} $$

In summary, the state equation of the offset error in the *x* direction can be expressed as:

17 $$ \left\{ {\begin{array}{*{20}l} {\dot{e}_{x1} = e_{x2} } \hfill \\ {\dot{e}_{x2} = \frac{{k_{ecc} }}{m}e_{x1} - \frac{1}{m}F_{cx} + \ddot{x}^{*} - \frac{1}{m}k_{ecc} x^{*} + \frac{1}{m}F_{xr} } \hfill \\ \end{array} } \right. $$

Similarly, the state equation of the offset error in the y direction can be obtained, that is:

18 $$ \left\{ {\begin{array}{*{20}l} {\dot{e}_{y1} = e_{y2} } \hfill \\ {\dot{e}_{y2} = \frac{{k_{ecc} }}{m}e_{y1} - \frac{1}{m}F_{cy} + \ddot{x}^{*} - \frac{1}{m}k_{ecc} y^{*} + \frac{1}{m}F_{yr} } \hfill \\ \end{array} } \right. $$

So far, the design of the torque sliding mode controller and suspension sliding mode controller of BPMSM has been completed.

### Parameter optimization of sliding mode controller with the GAPSO algorithm

When the conventional sliding mode control is applied to the motor control of the blood pump, it is easy to produce the phenomenon of system chattering. For this reason, this section proposes the torque sliding mode controller and the suspension sliding mode controller using the GAPSO algorithm for the bearingless permanent magnet sheet motor. The parameters are optimized to improve the control performance of the sliding mode controller.

The iterative formula of the traditional PSO is^23^:

19 $$ V_{p}^{k + 1} = \omega V_{p}^{k} + c_{1} r_{1} (W_{p} - X_{p}^{k} ) + c_{2} r_{2} (W_{g} - X_{p}^{k} ) $$

20 $$ X_{p}^{k + 1} = X_{p}^{k} + V_{p}^{k + 1} $$

where, $Xp$ and $Vp$ are the position and velocity of the p-th particle in the particle swarm respectively, $Wp$ is the position of the p-th particle in the particle swarm when it reaches the optimal position, $Wg$ represent the position when the entire particle swarm reaches the optimal position,$k$ is the number of iterations; $\omega$ is Particle inertia weight; $c_{1} ,\;c_{2}$ are the learning factors; $r_{1} ,\;r_{2}$ is a random number in [0 1].

**Figure 4:**
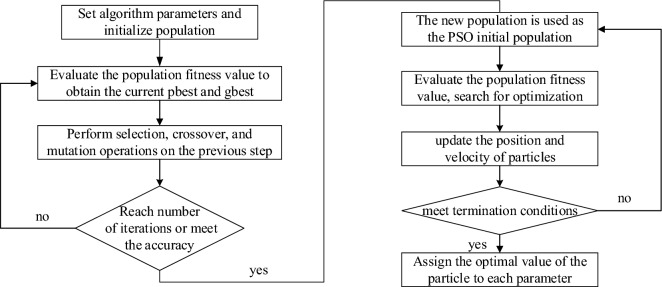
The optimization process of the GAPSO algorithm.

To improve the optimization performance of the PSO and improve its defects of poor convergence performance and easy to fall into local optimum, this paper introduces a improvement algorithm which integrating the genetic algorithm and the PSO^24^. Then, the GAPSO algorithm is applied to sliding mode controller, which can improve the performance of particle swarm optimization and improve the defects of poor convergence performance and ease to fall into local optimum. Figure 4 is the flowchart of the above improvement algorithm^7^.

**Figure 5:**
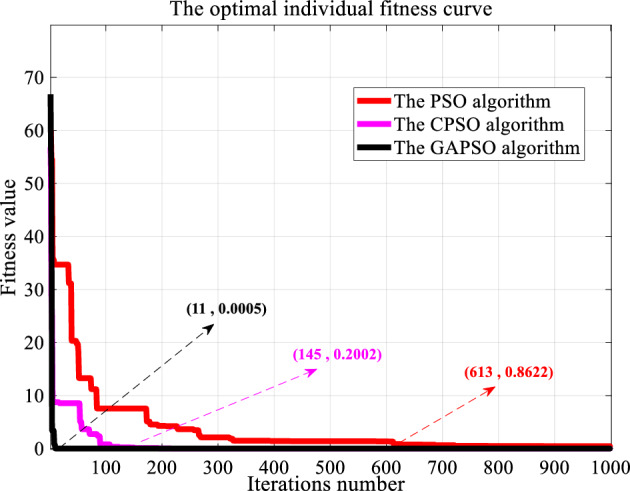
Convergence comparison of several kinds of PSO algorithms.

In our previous studies, the superiority of the GAPSO algorithm was simulated and verified^7^. At that time, several different particle swarm optimization algorithms were used to optimize commonly used test functions, and Fig. 5 shows the results of previous tests.

As shown in Fig. 5, it is easy to conclude that the convergence performance of the GAPSO algorithm is the best, and its efficiency is dozens of times that of the other two PSO algorithms, indicating that it is more flexible and less prone to falling into local optima. It can be seen that the GAPSO algorithm is the most suitable method to solve the PSO parameter optimization problem of sliding mode controller in the BPMSM. Therefore, this article uses it to optimize the parameters of the motor controller.

The parameter settings of the GAPSO algorithm in this article are shown in Table 1.

**Table 1:** The parameter settings of the GAPSO algorithm.

Parameter	Value	Parameter	Value
Initial population size *N*_*p*_	40	Selection probability *P*_*s*_	0.05
Inertia weight *w*	1.49	Cross probability *P*_*c*_	0.85
Acceleration factor *c*_1_	2	Variation probability *P*_*m*_	0.05
Acceleration factor *c*_2_	2	Iterations *G*	150

The integral time weighted absolute error (ITAE) is used as the optimization function, which has the advantage of generating small oscillations and overshoots. The expression of ITAE is as follows:

21 $$ J_{{{\text{ITAE}}}} = \int_{0}^{\infty } {t\left| {e(t)} \right|} dt $$

where, *e*(*t*) is the error between the expected output of the system and the actual output.

Next, the GAPSO algorithm was used to optimize the parameters of the sliding mode controller, and the optimization results are shown in Table 2.

**Table 2:** The operating conditions for which the controller was optimized.

Parameter	$\beta$	$q$	$p$	$l_{g}$	$\varepsilon$	$k$	$c$
Optimized value	1000	9	7	80	75	50	50

## Simulation study

**Figure 6:**
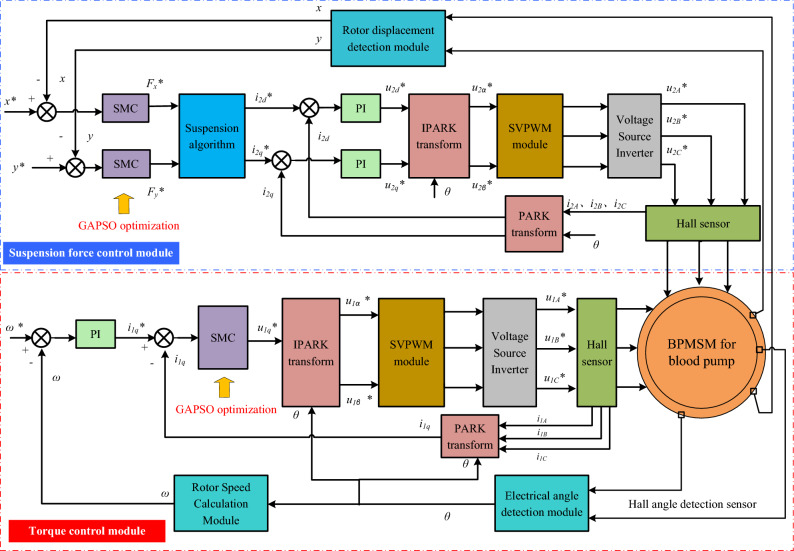
The block diagram of BPMSM sliding mode control system based on GAPSO optimization.

The control system of BPMSM for the blood pump is more complicated, including torque control subsystem and suspension control subsystem. The block diagram of the BPMSM sliding mode control system optimized based on the GAPSO algorithm is established in MATLAB/ Simulink, as shown in Fig. 6.

Because the rotation control part of the control system is basically consistent with the control of common permanent magnet synchronous motors, there will be no excessive description. Next, the suspension control section was described step-by-step.

Step 1.: As shown in Fig. 6, the actual offsets *x* and *y* measured by the displacement sensor rotor as feedback is compared with the reference offsets x* and y* , then the difference between them is input into a sliding mode controller optimized based on the GAPSO algorithm for calculation, to obtain the reference values *F*_x_* and *F*_y_* of the radial suspension force.
Step 2.: The suspension force reference value is converted into current reference values *i*_2d_* and *i*_2q_* through the suspension algorithm (force/current conversion module), and the difference is obtained by comparing it with the actual values *i*_2*d*_ and *i*_2*q*_ measured by the Hall sensor. Then, the voltage reference values *u*_2d_* and *u*_2q_* in the d-q coordinate system are calculated based on the difference.
Step 3.: After ipark transformation, the voltage reference values *u*_2α_* and *u*_2β_* in the stationary coordinate system are obtained. The obtained voltage reference values are then subjected to a space vector pulse width modulation module (SVPWM) to obtain three pairs of voltage control signals *u*_2A_*, *u*_2B_*, and *u*_2C_* for the voltage source inverter.
Step 4.: By controlling the amplitude and phase of the suspension force winding current through *u*_2A_*, *u*_2B_*, and *u*_2C_*, stable and active control of the suspension force is achieved, thereby stabilizing the rotor of the blood pump motor in the pump chamber.

In Fig. 6, ω*, x*, and y* are the rotational speed expected values, the expected displacement in the *x* direction, and the expected displacement in the *y* direction, respectively. *ω*, *x*, and *y* are the rotational speed real-time values, the real-time displacement in the *x* direction, and the real-time displacement in the *y* direction, respectively. *F*_x_* and *F*_y_* represent the expected levitation forces in the directions of *x* and *y* in *xy* coordinates, respectively. *i*_2d_* and *i*_2q_* respectively represent the expected levitation current in the directions of *d* and *q* in *dq* coordinates, respectively. *u*_2d_* and *u*_2q_* respectively represent the expected levitation voltage in the directions of *d* and *q* in *dq* coordinates, respectively. *u*_2α_* and *u*_2β_* respectively represent the expected levitation voltage in the directions of *α* and *β* in *αβ* coordinates, respectively. *u*_2A_*, *u*_2B_*, and *u*_2C_* respectively represent the expected levitation voltage in the directions of *A, B* and *C* in *ABC* coordinates, respectively. *i*_*2A*_, *i*_*2B*_, and *i*_*2C*_ respectively represent the real-time levitation current in the directions of *A, B* and *C* in *ABC* coordinates, respectively. *θ* represents the angular position of the rotor. The SMC is the sliding mode controller subsystem, the PI is the proportional integral controller, and the SVPWM is the space vector debugging subsystem. The GAPSO is a parameter optimization algorithm designed for SMC controllers. The PARK transform is a coordinate transformation from *abc* coordinates to *dq* coordinates, and the IPARK transform is the opposite of the former. The abbreviation in the torque subsystem is similar to the corresponding abbreviation in the suspension subsystem, so it is omitted here.

The simulation parameters of BPMSM for the blood pump are as follows: the number of pole pairs of the torque winding is 1, the number of pole pairs of the suspension winding is 2, the self-inductance of the torque winding is set to 8 mH, the leakage flux is set to 1 nH, the self-inductance of the suspension winding is set to 4.5 mH, the stator resistance is set to 2.875 Ω, the mass of the rotor is set to 0.15 kg, the moment of inertia is set to 5.6 × 10^–4^ kg m^2^, the target speed of the rotor is set to 1200 r/min, and the rotor speed in the *x* direction is The target eccentric displacement is set to − 0.2 mm, the target eccentric displacement in the *y* direction is set to 0.15 mm, the equivalent flux linkage of the rotor is set to 0.125 Wb, and the simulation time is set to 1 s.

To verify the suspension and rotation performance of the SMC controller after rotor disturbance, external disturbances were added during the simulation process. On the one hand, after simulating the stable motion of the rotor at the predetermined speed, a torque of 2N. m is applied in the opposite direction of the rotor rotation at 0.3 s. On the other hand, after the simulated rotor reaches the equilibrium position in radial suspension, a disturbance force is applied radially, with a disturbance force of 10N in the x direction and -5N in the y direction.

**Figure 7:**
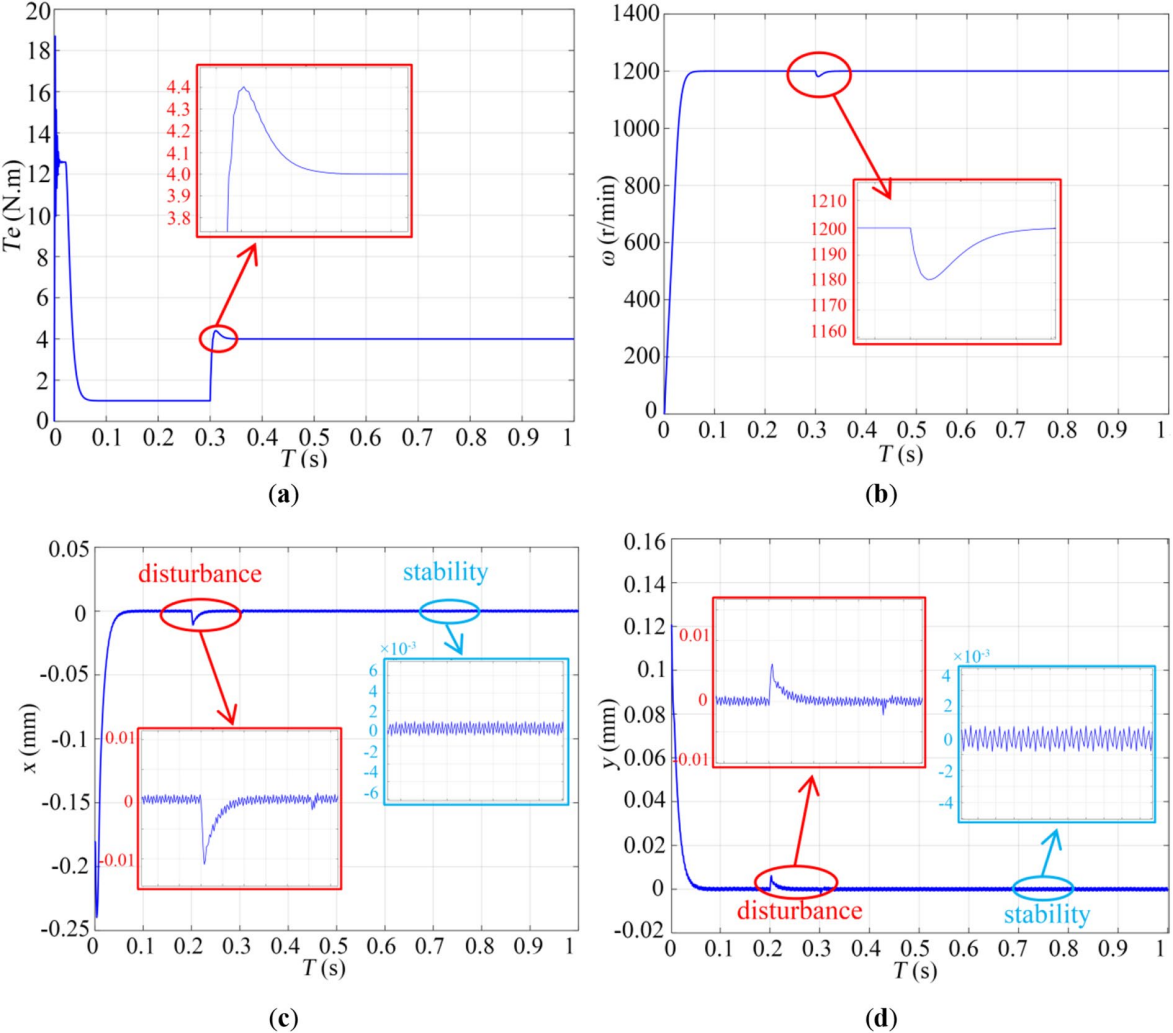
The simulation result: (**a**) torque; (**b**) rotor speed; (**c**) offset in *x* direction; (**d**) offset in *y* direction.

After setting all parameters, the Simulink simulation is performed. During the simulation, the GAPSO algorithm will first optimize the performance parameters of the sliding mode controller, then assign the optimal parameters to the sliding mode control system, and finally, obtain the dynamic characteristic curve of the control system. The results are shown in Fig. 7.

Figure 7a is the BPMSM electromagnetic torque output curve. The maximum electromagnetic torque of the motor is 12.5 Nm at the starting point. At 0.3 s, the sudden curve change is due to an added disturbance. The increased torque is to balance the effects of the disturbance. Figure 7b is the BPMSM rotor speed output curve. The rotor speed rises rapidly to the given speed value at 0–0.07 s, then the speed tends to be stable, and the overshoot is controlled within the range of 0.5%. The steady-state error of the rotational speed is less than 2.5 r/min, and the disturbance has little effect on the rotational speed. It is worth mentioning that only a small amplitude oscillation occurs and then returns to the equilibrium position quickly, proving that the motor has good dynamic acceleration performance.

Figure 7c and d are the curves of the eccentric displacement of the rotor in the *x* and *y* directions with time under the action of the suspension winding, respectively. From Fig. 7c, the response speed of the rotor suspension control system is breakneck. After 0.05 s, the curve reaches the equilibrium position. The sudden change at 0.2 s in the figure is due to an added disturbance force in the radial direction. As shown in Fig. 7d, after being disturbed by force, the maximum offset of the rotor in the *x* directions and *y* directions is only 0.015 mm and 0.005 mm. It returns to the equilibrium position after 0.03 s, and it has maintained stability since then. Furthermore, combined with Fig. 7c and d, the effect of the torque disturbance at 0.3 s on the eccentric displacement in the *x* and *y* directions is mainly negligible. It can be concluded that the control system of BPMSM has better dynamic suspension performance. The above results verify that the GAPSO-based BPMSM sliding mode control method designed in this paper is effective.

**Figure 8:**
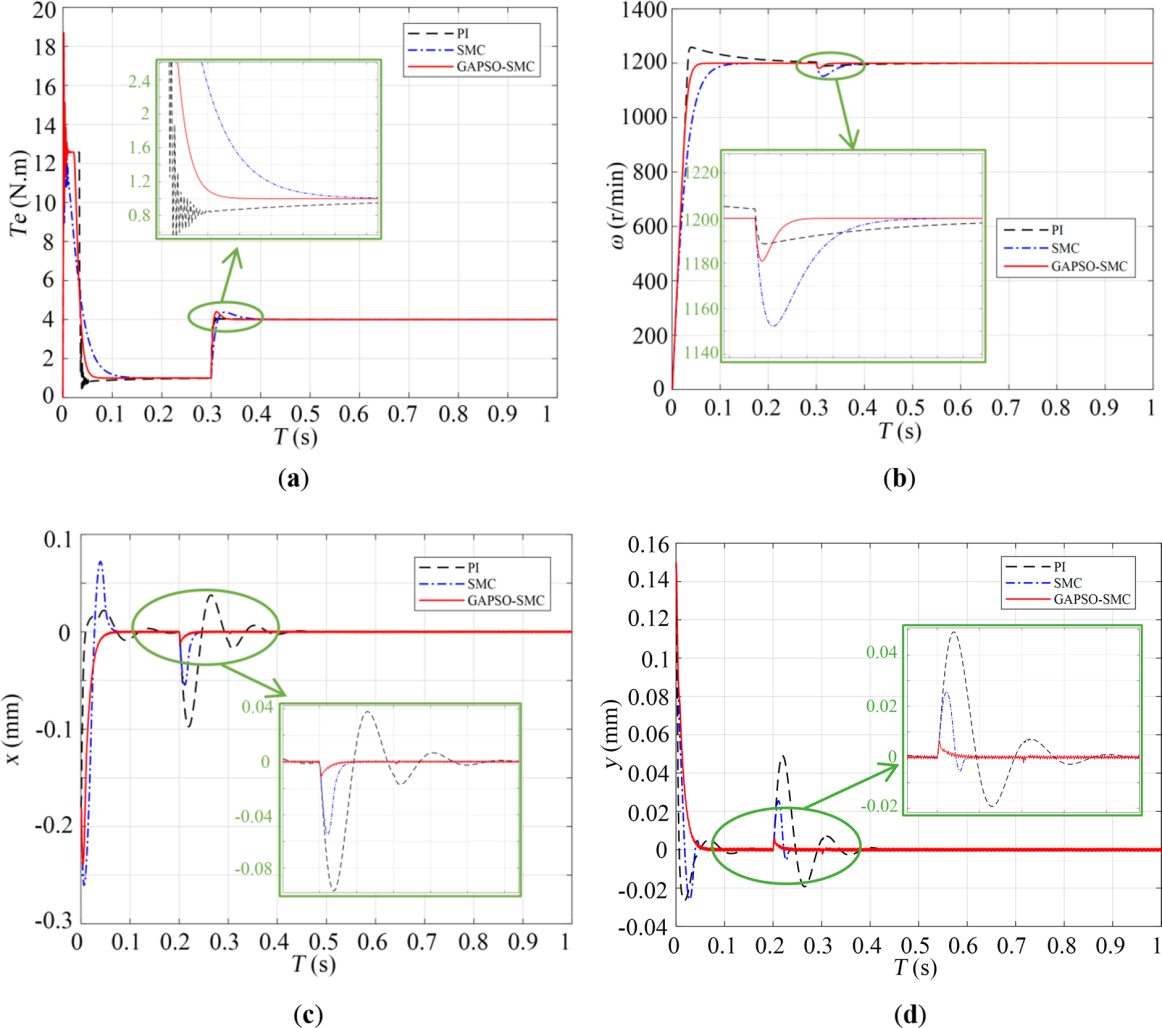
The comparison diagram of the three control methods: (a) torque; (b) rotor speed; (c) offset in *x* direction; (d) offset in *y* direction.

To further highlight the superiority of the design method, we established a three-block diagram of the BPMSM control system, which is traditional PID control, conventional sliding mode control, and the improved sliding mode control method, respectively, in Simulink. And comparative simulation analysis is carried out. The comparison results of the three methods on torque, rotational speed and radial offset are shown in Fig. 8.

Figure 8a show that the torque curve of two sliding mode controls are more smooth than the torque curve of the PI control. Moreover, PI control may exhibit unstable oscillations in its torque during startup and when subjected to external interference, which is detrimental to the motor. In terms of the rotor speed performance, Fig. 8b show that compared with the maximum overshoot of rotor speed of the PI control, the maximum overshoot of rotor speed of the improved sliding mode control and the ordinary sliding mode control is reduced by 67.2% and 17.2%, respectively. The above analysis indicates that sliding mode control is far superior to PI control in terms of stability. In addition, compared with the ordinary sliding mode control, the response time of rotor speed of the improved sliding mode control is 61.6% faster, the response time is 74.3% faster, and the fluctuation is significantly reduced, which proves that the parameter optimization effect of GAPSO algorithm is effective. The motor has good electromagnetic torque performance and rotor speed performance under this control method.

In terms of suspension control performance, Fig. 8c and d show that compared with PI control and ordinary sliding mode control, the maximum offset of the new method in the *x* direction is reduced by 87.5% and 76.2%, the maximum offset in the *y* direction is reduced by 87.5% and 76.2%, respectively. The above data show that the new method proposed in this paper is more stable. After adding the disturbance force, the curve oscillation under the improved sliding mode control system is minimized compared with the other two control methods, and realized the stable suspension of the rotor in the radial direction. Therefore, the sliding mode control based on GAPSO outperforms the PI control and the ordinary sliding mode control in response to overshoot, system stability, and resistance to external disturbances.

From the above analysis, it can be concluded that the control method in this paper has excellent advantages in torque acceleration dynamic performance and suspension control dynamic performance.

## Simulation experiments

Aortic blood flow is an important parameter when the blood pump is runningand an important indicator of whether the blood pump can meet the blood perfusion of the human body. The flow of blood fluid into an organ or tissue through blood circulation system is known as perfusion. The diac output blood flow in normal adult males was 4.5–6.0 L/min. The joins of human speed and blood flow are supposed to verify the effectiveness of the control method in this paper in controlling the blood flow of the blood pump. Based on the control method designed in "Mathematical model of the BPMSM" section, by controlling the speed of the motor, adjust the blood flow, so that the blood flow can meet the demand at any time.

**Figure 9:**
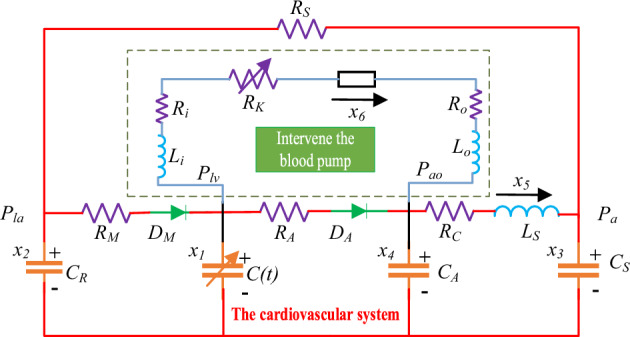
The schematic diagram of coupling model.

To verify the control effect of the design method in "Mathematical model of the BPMSM" section and its performance when applied to blood pump, it is necessary to establish a coupled model of the cardiovascular circulatory system and heart pump, and then carry out simulation work based on the model. The coupling mode between the cardiovascular circulatory system and the heart pump is shown in Fig. 9^24^. Table 3 lists the six variables and their associated physiological relevance in the model. Table 4 lists all model parameters and their associated physiological relevance.

**Table 3:** State variables in the combined cardiovascular system model.

Variable	Parameter	Physiological significance (unit)
*x*_1_(*t*)	*P*_*lv*_	Left ventricular pressure (mmHg)
*x*_2_(*t*)	*P*_*la*_	Left atrial pressure (mmHg)
*x*_3_(*t*)	*P*_*a*_	Arterial pressure (mmHg)
*x*_4_(*t*)	*P*_*ao*_	Aortic pressure (mmHg)
*x*_5_(*t*)	*Q*_*a*_	Blood flow (mL/s)
*x*_6_(*t*)	*Q*_*pump*_	Blood pump flow rate (mL/s)

**Table 4:** Variable values.

Parameter category and unit	Parameter	Physiological significance	value
blood viscosity resistance (mmHg s/ml)	*R*_*M*_	Mitral valve resistance	0.0050
	*R*_*A*_	Aortic valve resistance	0.0010
	*R*_*C*_	Characteristic resistance	0.03980
	*R*_*S*_	Systemic vascular resistance	1.0000
Casing resistance (mmHg s/ml)	*R*_*i*_	Casing inlet resistance	0.0677
	*R*_*o*_	Casing outlet resistance	0.0677
	*R*_*P*_	Differential pressure parameter	0.1707
	*R*_*k*_	Suction resistance	0 (*x*_1_ > 1) or − 3.5(*x*_1_ − 1) (*x*_1_ ≤ 1)
Compliance (ml/mmHg)	*C*_*R*_	Left atrial compliance	4.4000
	*C*_*S*_	Systemic compliance	1.3300
	*C*_*A*_	Aortic compliance	0.0800
Blood inertia (mmHg s^2^/ml)	*L*_*S*_	Aortic blood inertia	0.0005
Casing inertia (mmHg s^2^/ml)	*L*_*i*_	Casing inlet inertia	0.0127
	*L*_*o*_	Casing outlet inertia	0.0127
	*Lp*	Export difference parameters	0.02177
Time-varying parameter	*C(t)*	Ventricular elasticity function	time-varying
	*E(t)*	Left ventricle compliance	
Elastic variable (mmHg/ml)	*E*_*max*_	Myocardial contractility	2.000 (normal heart)
			1.000 (heart failure)
	*E*_*min*_		0.060

From Fig. 9, The paths from left to right (*x*_2_ to *x*_3_) in the lower half represent the left atrium, left ventricle, aorta, and artery, respectively. The path through *x*_6_ represents the blood flow through the blood pump. Among them: *C*_*R*_ and *C*(*t*) represent the compliance of the left atrium and left ventricle. The compliance is used to characterize the degree to which the vascular volume changes with blood pressure. The diodes *D*_*M*_ and *D*_*A*_ represent the mitral and aortic valves. They are on and off and represent valve opening and closing, respectively. *R*_*M*_ and *R*_*A*_ are mitral and aortic valve flow resistance, respectively. *L*_*S*_ represents the inertia of blood. *R*_*C*_ and *C*_*S*_ denote peripheral resistance and arterial compliance, respectively. The time-varying of this coupled model is the volume characteristic of the left ventricle, reflected by a time-varying capacitance value *C(t)*, which is the reciprocal of the ventricular elasticity function *E(t)*.

**Figure 10:**
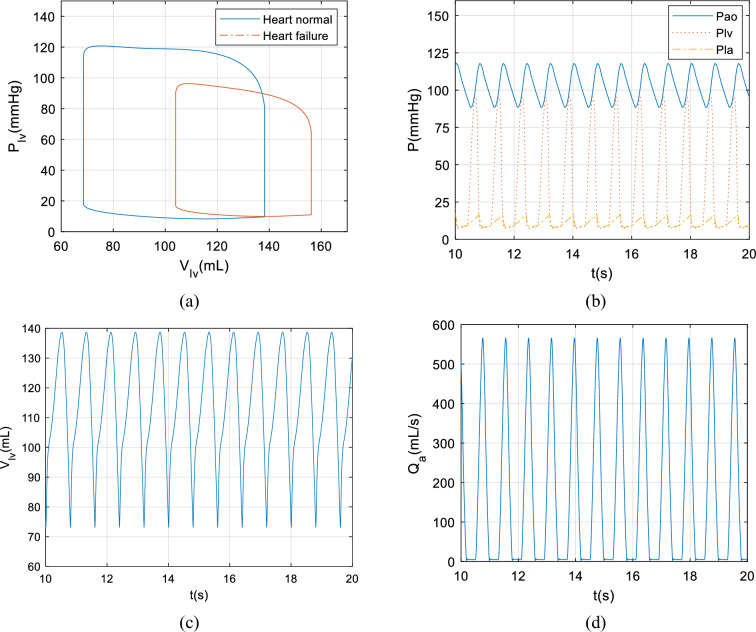
The haemodynamic simulation results: (**a**) the *PV-loop*, which is used to characterize the process of pressure and volume of the cardiac cycle.; (**b**) the pressure curve, including *P*_*ao*_, *P*_*lv*_, and *P*_*la*_; (**c**) the left ventricular volume curve; (**d**) the aortic blood flow curve.

The control system simulation is conducted in MATLAB/Simulink software, and the above model is built in Simulink. The SMC control algorithm is written as an m function and imported into the coupling model. The sampling period is set to 0.001 s, and the simulation time is set to 30 s. The elastic size of the left ventricular end-systolic and left ventricular end-diastolic in the model were modified to complete the setting of heart failure. At the same time, the blood pump was added to assist in pumping blood from the failed heart, whose model is written as an m function and imported into the coupling model. The hemodynamic curves hemodynamic indicators such as blood pressure, blood flow, and left ventricular volume over time can be obtained by running the simulation. The results are shown in Fig. 10.

Figure 10a shows the simulation results of the *PV-loop* comparison between normal and failed hearts. Both normal and failed heart blood pressure are consistent with normal clinical results, indicating that the coupled model is successful and can simulate cardiovascular characteristics. From Fig. 10a, it can be observed that after heart failure, its blood pressure and pump blood flow both decrease significantly, which can lead to insufficient pump blood. At this point, it is necessary to intervene in the blood pump to assist in pumping blood.

From Fig. 10b, it is easy to know that after using the blood pump with a speed of 5000 r/min to assist in pumping blood, the aortic pressure can be maintained within the range of 87 mmHg to 118 mmHg, indicating that the blood pump can provide sufficient blood pressure to support pump blood. In addition, the numerical results of left ventricular pressure, venous pressure, and aortic pressure in the Fig. 10b are very close to the physiological range of normal human, indicating that the coupling model is effective and consistent with the normal cardiovascular system.

Figure 10c and d shows the change in the left ventricular volume and the aortic blood flow with time. It can be calculated that the peak aortic blood flow is as high as 562.3 mL, indicating that the energy of blood flowing out of the aorta with the assistance of a blood pump is strong, which can deliver the heart's blood bed to every corner of the body of patients with heart failure. In addition, the stroke volume of the heart pump is 66.17 mL, and the cardiac output is 4.89 L/min. These two values are within the physiological value range (4.5–6.0 L/min) of normal people, indicating that the improved sliding mode control can assist in pumping blood sufficiently, thus meeting the need for systemic blood perfusion.

### Consent to participate

Informed consent was obtained from all individual participants included in the study.

## Conclusion

The third generation of blood pump aims to stably suspend the rotor impeller in the pump room and assist the natural cardiac blood beating in meeting the blood perfusion needs of the human circulation system. The sliding mode control method optimized based on the GAPSO algorithm has achieved good results in the blood pump speed, suspension, and blood flow control. The results show that, compared with the PI control and the ordinary sliding mode control, our method has greatly improved the control accuracy, response speed, and stability of the control system, and significantly improved the control performance of the BPMSM. At the same time, the blood flow is controlled by controlling the rotational speed of the motor used in the blood pump to verify the effectiveness of our control method in controlling the blood flow of the blood pump.

In future research, On the one hand, further experimental research will be carried out after the research group's blood pump prototype is developed. On the other hand, continue to carry out the research work on improving the pulsatility of the blood pump by the BPMSM control system. By optimizing the control system, the pumping characteristics and laws of the blood pump are more in line with the natural physiological activity characteristics of the human body, improving the recovery speed of patients and reducing the complications from the blood pump.

## Data Availability

I do not wish to share my data, because all data generated or analysed during this study are included in this published article.
